# Facile synthesis of flower like copper oxide and their application to hydrogen peroxide and nitrite sensing

**DOI:** 10.1186/1752-153X-5-75

**Published:** 2011-12-02

**Authors:** Li Zhang, Feifei Yuan, Xiaohu Zhang, Liming Yang

**Affiliations:** 1 College of Chemistry and Materials Science, Anhui Key Laboratory of Functional Molecular Solids, Anhui Normal University, Wuhu 241000, P. R. China

## Abstract

**Background:**

The detection of hydrogen peroxide (H_2_O_2_) and nitrite ion (${\text{NO}}_{2}^{-}$) is of great important in various fields including clinic, food, pharmaceutical and environmental analyses. Compared with many methods that have been developed for the determination of them, the electrochemical detection method has attracted much attention. In recent years, with the development of nanotechnology, many kinds of micro/nano-scale materials have been used in the construction of electrochemical biosensors because of their unique and particular properties. Among these catalysts, copper oxide (CuO), as a well known p-type semiconductor, has gained increasing attention not only for its unique properties but also for its applications in many fields such as gas sensors, photocatalyst and electrochemistry sensors. Continuing our previous investigations on transition-metal oxide including cuprous oxide and α-Fe_2_O_3 _modified electrode, in the present paper we examine the electrochemical and electrocatalytical behavior of flower like copper oxide modified glass carbon electrodes (CuO/GCE).

**Results:**

Flower like copper oxide (CuO) composed of many nanoflake was synthesized by a simple hydrothermal reaction and characterized using field-emission scanning electron microscopy (FE-SEM) and X-ray diffraction (XRD). CuO modified glass carbon electrode (CuO/GCE) was fabricated and characterized electrochemically. A highly sensitive method for the rapid amperometric detection of hydrogen peroxide (H_2_O_2_) and nitrite (${\text{NO}}_{2}^{-}$) was reported.

**Conclusions:**

Due to the large specific surface area and inner characteristic of the flower like CuO, the resulting electrode show excellent electrocatalytic reduction for H_2_O_2 _and oxidation of ${\text{NO}}_{2}^{-}$. Its sensitivity, low detection limit, fast response time and simplicity are satisfactory. Furthermore, this synthetic approach can also be applied for the synthesis of other inorganic oxides with improved performances and they can also be extended to construct other micro/nano-structured functional surfaces.

## Background

The detection of hydrogen peroxide (H_2_O_2_) is of great important in various fields including clinic, food, pharmaceutical and environmental analyses, because H_2_O_2 _is a chemical threat to the environment and the production of enzymatic reactions [1]. Many methods have been developed for the determination of H_2_O_2_, such as titrimetry [2], spectrophotometry [3], chromatography [4] and chemiluminescence [5]. Compared with the above detection methods, the electrochemical detection of H_2_O_2 _was introduced to achieve a low detection limit and a low cost compared with the other detection methods. In recent years, with the development of nanotechnology, many kinds of micro/nano-scale materials have been used in the construction of electrochemical biosensors because of their unique and particular properties. A number of excellent reports have focused on the electrochemical determination of H_2_O_2 _utilizing noble metals including gold [6], silver [7], platinum [8,9], palladium [10], and graphene-Pt nanoparticle hybrid material [11], transition metals and their oxides or complexes, such as MnO_2 _[12], Mn-nitrilotriacetate acid nanowires [13], CuO nanoparticles [14], Cu-Ni(OH)_2 _[15], layered double hydroxide [16], prussian blue [17], conducting polymers [18] and nanocomposite MnO_2_/MWNTs, Ag/GO [19,20], as well as enzyme and protein modified electrodes [21,22]. The low detection limit achieved with such platforms especially metal nanoparticle based electrodes is due to the enhancement in the signal-to-noise (S/N) ratio and increased mass transport to the electrode surface [11,21]. Although some chemically modified electrodes have been proposed to reduce the large overpotential required for the direct oxidation or reduction of H_2_O_2_, it is still interesting to develop new materials with high efficiency and small dimensions for the detection of H_2_O_2_.

Like H_2_O_2_, nitrite ion (${\text{NO}}_{2}^{-}$) is another often studied analyte in various fields including clinic, food, and environmental analyses because its excess level in the blood has been proved to lead to haemoglobin oxidation [23-26]. Also it may interact in the stomach with amines and amides forming highly carcinogenic N-nitrosamine, many of which are known to be carcinogens [27,28]. Therefore, ${\text{NO}}_{2}^{-}$ determination is important for environment security and public health. Although ${\text{NO}}_{2}^{-}$ is electroactive at carbon electrodes, its oxidation requires undesirably high overpotential and the voltammetric determination of it suffers from interference from other compounds. In order to improve the selectivity of ${\text{NO}}_{2}^{-}$ sensor, the operating potential should be efficiently lowered. Modified electrodes with suitable electro-catalysts on the surface of carbon electrodes can achieve the purpose with an improved oxidation response of ${\text{NO}}_{2}^{-}$[29-31].

Metal oxide electrodes possess some unique electrochemical properties compared to metal ones. Their advantages include enhancement of reaction rate due to redox couples of oxide species of two different states, as well as weak adsorption or complete exclusion of hydrogen species on an oxide surface [31]. Among these catalysts, copper oxide (CuO), as a well known p-type semiconductor, has gained increasing attention not only for its unique properties but also for its applications in many fields such as gas sensors, photocatalyst and electrochemistry sensors [30,32-36]. Continuing our previous investigations on transition-metal oxide including cuprous oxide and α-Fe_2_O_3 _[37,38] modified electrode, in the present paper we examine the electrochemical and electrocatalytical behavior of flower like copper oxide modified glass carbon electrodes (CuO/GCE). CuO was characterized by Powder X-ray diffraction (XRD), Field emission scanning electron microscopes (SEM) and cyclic voltammetry (CV) measurements. The electrochemical properties of the modified electrode were evaluated with regards to electrocatalytical reduction of H_2_O_2 _and electrocatalytical oxidation of ${\text{NO}}_{2}^{-}$.

## Experimental

### Reagents and apparatus

Cu(NO_3_)_2_·3H_2_O, NH_3_·H_2_O and hexamethylenetetramine was purchased from Shanghai Chemical Reagent Factory (Shanghai, China). NaNO_2 _and 30% H_2_O_2 _solution was purchased from Beijing Chemical Reagent Factory (Beijing, China). All of the other chemicals used were analytical grade and used without further purification. Double-distilled water was used for preparation of buffer and standard solutions. All solutions were purged with high-purity nitrogen for at least 30 min to remove oxygen. NaNO_2 _and H_2_O_2 _solution was diluted daily before the electrochemical measurements.

Electrochemical experiments were performed with CHI 440a electrochemical analyzer (ChenHua Instruments Co. Ltd., Shanghai, China) with a conventional three-electrode cell. The CuO/GCE, an Ag/AgCl and a platinum electrode was used as the working electrode, the reference and the auxiliary electrode, respectively.

### Synthesis of flower like CuO

In a typical synthesis, 0.7345 g Cu(NO_3_)_2_·3H_2_O and 0.8230 g hexamethylenetetramine (HMT) were dissolved into 25 ml distilled water under magnetic stirring. After 5.0 ml NH_3_•H_2_O (5%) was introduced into the mixture under stirring, the clear solution was transferred into a Teflon-lined steel-stainless autoclave of 40 ml. The autoclave was allowed to cool down to room temperature naturally after the system had been hydrothermally treated at 160°C for 6 h. Black precipitates were collected, washed with distilled water and ethanol several times to remove impurities. Finally, the precipitates were dried in air at 50°C for 6 h.

### Characterization of the samples

Powder X-ray diffraction (XRD) of the product was carried out on a Shimadzu XRD-6000 X-ray diffractometer equipped with Cu K*α *radiation (*λ *= 0.154060 nm), employing a scanning rate of 0.02°s^-1 ^and 2θ ranges from 20° to 70°. Field emission scanning electron microscopes (SEM) was obtained by JEOL JSM-6700 FESEM (operating at 10 kV).

### Electrode modification

The dispersed flower like CuO on the electrode were fabricated by the following way: Firstly, the glass carbon electrode (GCE, Φ = 3 mm) was polished with a 1700# diamond paper and washed successively with double distilled water and ethanol in an ultrasonic bath, then 15 cyclic scans were carried out in the potential of 2.0 to -2.0 V (vs. SCE) in the solution of 1.0 mol/l H_2_SO_4_. Secondly, 4 mg CuO was dispersed in 2 ml ethanol solution. Then 20 μl of CuO solution (2 mg/ml) was cast on the surface of GCE and dried in air. Thus flower like CuO modified GCE was obtained.

## Results and discussion

### Structures and morphology characterization

**Figure 1:**
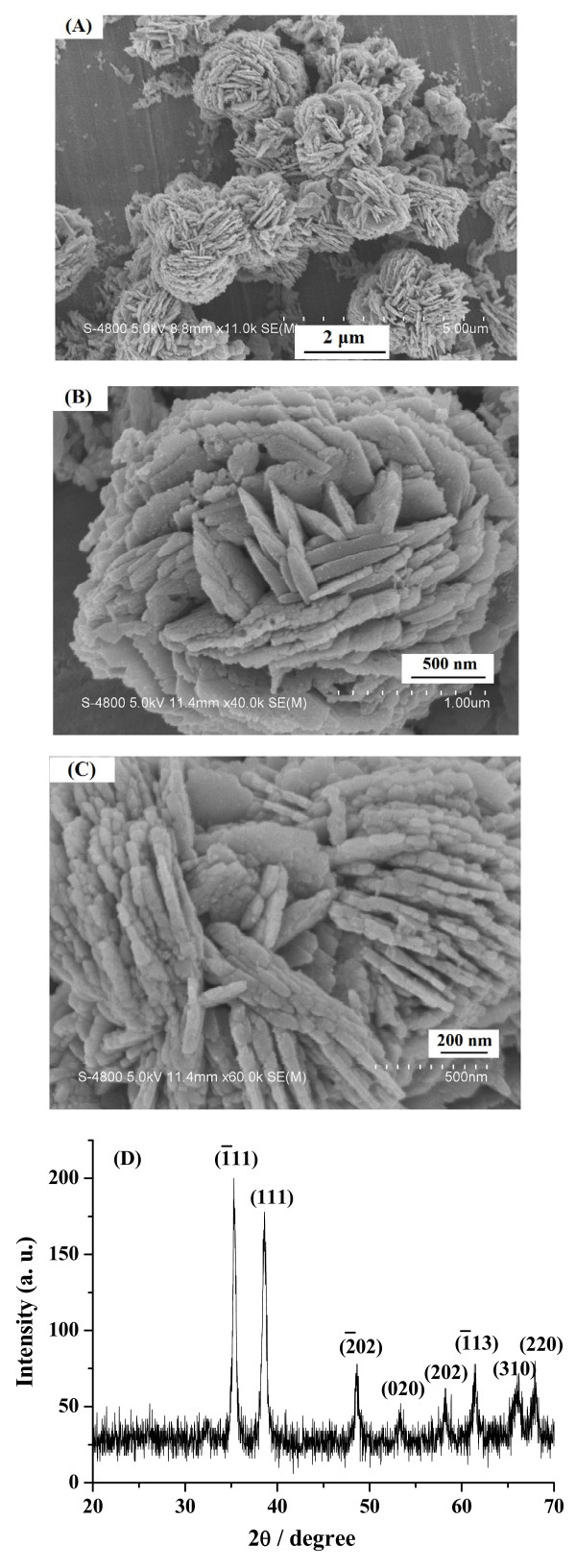
**(A, B, C) SEM image and (D) the XRD pattern of the product**.

The morphology and micro-structure of the product were investigated by SEM. Figure 1(A) and 1(B, C) are the SEM images of the sample. The low-magnification SEM image reveals that a large number of flower like CuO (Figure 1A) formed, composed of many nanoflake with average thickness of about 40 nm (Figure 1B, C), which may bring a favorite electronic property. Figure 1(C) shows the typical XRD patterns of the as-synthesized products All diffractions can be indexed as monoclinic phased CuO by comparison with JCPDS card files No. 48-1548 (a = 4.62 Å, b = 3.43 Å, and c = 5.06 Å). No characteristic peaks of other impurities were detected.

### Electrochemical property of flower like CuO modified GCE

**Figure 2:**
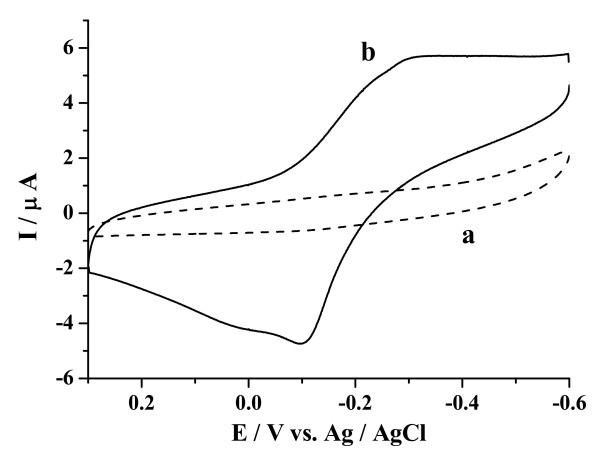
**Cyclic voltammograms (CVs) recorded in an oxygen-free 0.1 M PBS (pH 7.0) at the scan rate of 0.05 V/s, using various modified electrodes: (a) bare GCE and (b) CuO/GCE**.

Figure 2 shows the cyclic voltammetric responses of various electrodes in an oxygen-free 0.1 M phosphate buffer solution (PBS, pH 7.0). No redox peak was observed when the bare GCE was used as the work electrode (Figure 2a). While the CuO/GCE was employed as the work electrode, a pair of well-defined redox peaks was obtained (Figure 2b) with an average formal potential of -0.1895 V. The cathodic peak and anodic peak were -0.2820 V and -0.0970 V at a scan rate of 0.05 V/s, respectively. Obviously, the redox peaks were attributed to the electrochemical reaction of CuO. According to earlier reports [39], the cathodic peak corresponds to the reduction of CuO/Cu_2_O redox couple, and the anodic peak comes from the formation of CuO layer.

### Electroreduction behavior and amperometric response of H_2_O_2 _on the CuO/GCE

**Figure 3:**
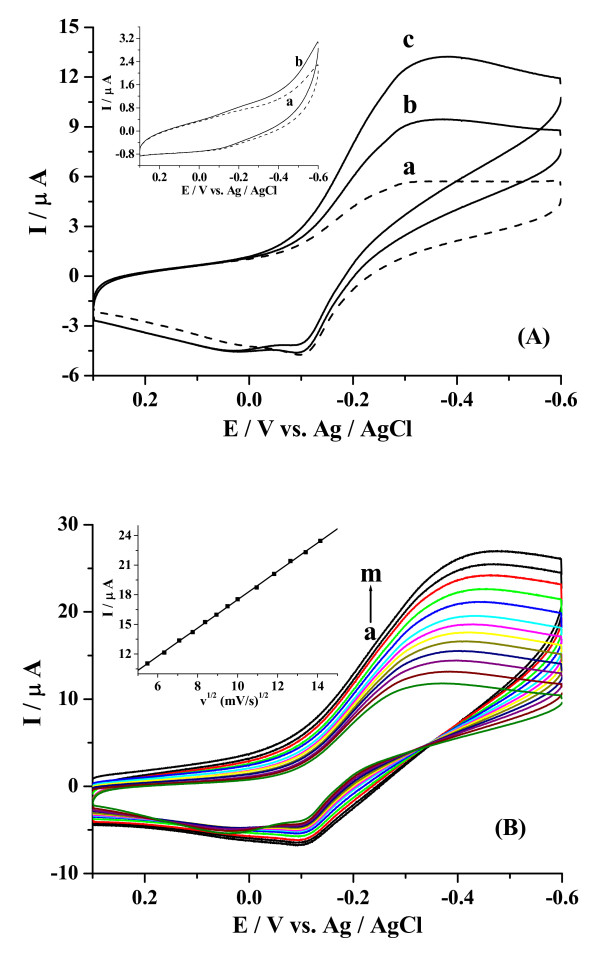
**(A) CVs of H_2_O_2 _in 0.1 M PBS of pH 7.0 for CuO/GCE in various concentrations: (a) 0 mM, (b) 1 mM, (c) 2 mM at scan rate of 50 mV/s**. Inset: CVs of H_2_O_2 _at bare GCE in various concentrations: (a) 0 mM, (b) 1 mM. (B) CVs of 2 mM H_2_O_2 _at the CuO/GCE in deoxygenized 0.1 M PBS (pH 7.0) at different scan rates (from inside to outside: a → m: 0.03, 0.04, 0.05, 0.06, 0.07, 0.08, 0.09, 0.10, 0.12, 0.14, 0.16, 0.18, 0.20 V/s, respectively). The linear dependence of peak current with the square root of scan rate was shown in the inset.

Figure 3(A) shows the cyclic voltammograms of the CuO/GCE in the absence and presence of H_2_O_2_. When H_2_O_2 _was added to the pH 7.0 PBS, compared with the system with no H_2_O_2 _present (a), an obvious increase of the reduction peak was observed in deoxygenized environment (b, c). However, no electrochemical reduction peak was observed when the cyclic voltammetric scan was performed at bare GCE under the same conditions (Inset in Figure 3). The experimental results indicated that the CuO/GCE exhibited excellent electrocatalytic activity to H_2_O_2_. Regarding the reduction peaks of H_2_O_2 _at CuO/GCE, the effect of potential scan rate was investigated clearly. As can been seen from Figure 3(B), the reduction peak current is proportional to the square root of scan rate in the range of 30-200 mV/s, i_pa_/μA = 3.193 + 1.431 v^1/2^/mV·s^-1^, R = 0.9998, indicating a diffusion controlled process [40].

The catalytic mechanism for the reduction of H_2_O_2 _can be assumed as Cu (II) was first electrochemically reduced to Cu (I), which reacted chemically with H_2_O_2 _and resulted in the conversion of H_2_O_2 _to H_2_O and in the regeneration of the catalyst [14].

**Figure 4:**
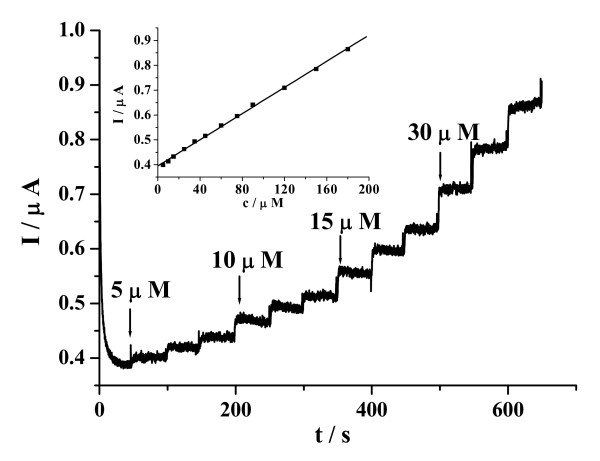
**Amperometric responses of CuO/GCE upon the successive addition of H_2_O_2 _into gently stirred 0.1 M PBS at -0.20 V**. Inset: the linear relationships between the catalytic current and the concentration.

Figure 4 illustrates current-time plots for the CuO/GCE with successive step changes of H_2_O_2 _concentration. As the H_2_O_2 _was injected into the stirring PBS, the steady-state currents reached another steady-state value (95% of the maximum) in less than 3 s. Such a fast response implies that the CuO can promote the oxidation of H_2_O_2_. The linear relationship between the catalytic current and the concentration is shown in the inset of the Figure 4. As can be seen, the CuO/GCE displays linear response range of 5.0 × 10^-6 ^to 180.0 ×10^-6 ^M (correlation coefficient: 0.9993), with a detection limit of 1.6 × 10^-6 ^M at a signal-to-noise ratio of 3, which is comparable or lower than detection limits obtained with protein [22,41] or Ag nanoparticle [42,43] based electrochemical sensors. We have summarized various H_2_O_2 _sensors in Table 1 with respect to the linear range and the detection limit. It can be seen that the performance of the noble metal Pt nanoparticle based sensor [9,11,21] is excellent compared to the other material based electrode with respect to the linear range and the detection limit. Nevertheless, it should be noted that the detection limit achieved with the CuO/GCE sensor was comparable or lower than that of protein or Ag nanoparticle based electrodes [41-43], and further CuO was prepared by a simple hydrothermal reaction and didn't require diffusional redox mediator and enzyme loading.

**Table 1:** Comparison of performances of different electrochemical sensors for hydrogen peroxide.

Electrode	Linear range	Detection limit	Reference
Pt-nanoparticle GCE	0.5 nM-4 mM	0.5 nM	[9]
Graphene-Pt nanoparticle-GCE	0.5-12 mM	0.5 nM	[11]
Enzyme integrated silicate-Pt nanoparticle		0.5 nM	[21]
Redox protein based EC	0.25-50 μM	0.25 μM	[22]
Hb-kieselgubr EC	5.0-300 μM	2.1 μM	[41]
Ag microspheres-GCE	0.2-2.0 mM	1.2 μM	[42]
Ag-nano-DNA GCE	4.0 μM -16.0 mM	1.7 μM	[43]
CuO-GCE	5.0-180.0 μM	1.6 μM	This work

### Electrooxidation behavior and amperometric response of ${\text{NO}}_{2}^{-}$ on the CuO/GCE

**Figure 5:**
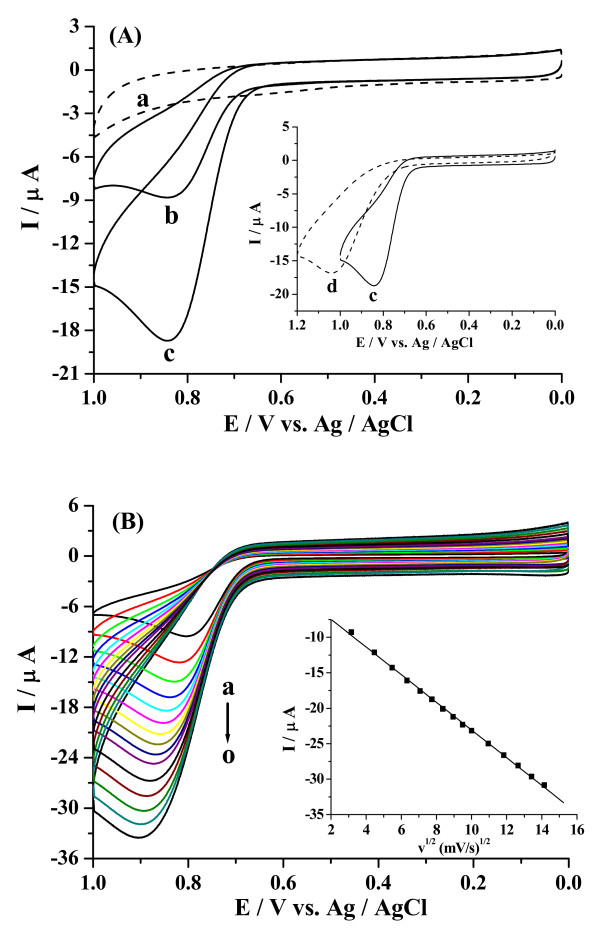
**(A) CVs of ${\text{NO}}_{2}^{-}$ in 0.1 M PBS of pH 7.0 for CuO/GCE in various concentrations: (a) 0 mM, (b) 0.5 mM, (c) 1 mM at the scan rate of 50 mV/s**. Inset: CVs of 1 mM ${\text{NO}}_{2}^{-}$ at CuO/GCE (c) and bare GCE (d) in the same condition. (B) CVs of 1 mM ${\text{NO}}_{2}^{-}$ at the CuO/GCE in deoxygenized 0.1 M PBS (pH 7.0) at different scan rates (from inside to outside: a → o: 0.01, 0.02, 0.03, 0.04, 0.05, 0.06, 0.07, 0.08, 0.09, 0.10, 0.12, 0.14, 0.16, 0.18, 0.20 V/s, respectively). The linear dependence of peak current with the square root of scan rate was shown in the inset.

Figure 5(A) shows typical CVs of the CuO/GCE in the absence and presence of ${\text{NO}}_{2}^{-}$, at the scan rate of 0.05 V/s in the oxygen-free 0.1 M PBS (pH 7.0). A well-defined nitrite oxidation peak could be observed at ca. + 0.843 V vs. Ag/AgCl (Figure 5(A) b, c), which is corresponding to the convertion of ${\text{NO}}_{2}^{-}$ to ${\text{NO}}_{3}^{-}$ through a two-electron oxidation process [44]. In contrast, no peak was observed when cyclic voltammogram of the same electrode was run in 0.1 M phosphate buffer solution (PBS) (Figure 5(A) a). Cyclic voltammogram of the bare GCE was also run in the 1 mM ${\text{NO}}_{2}^{-}$ solution. The peak corresponding to the oxidation of nitrite appears at ca. +1.038 V vs. Ag/AgCl (Figure 5(A) inset d), 195 mV more positive potential than the one obtained for modified GCE, also being of smaller intensity. This result might be explained by CuO film providing abundant active sites improving the electrocatalytic activity for ${\text{NO}}_{2}^{-}$. The catalytic oxidation mechanism can be explained with the following process:

(1) $${\text{NO}}_{2}^{-}↔2\text{N}{\text{O}}_{2}+2{\text{e}}^{-}$$

(2) $$2\text{N}{\text{O}}_{2}+{\text{H}}_{2}\text{O}\to {\text{NO}}_{3}^{-}+{\text{NO}}_{2}^{-}+2{\text{H}}^{\text{ + }}$$

(3) $${\text{NO}}_{2}^{-}+{\text{H}}_{2}\text{O}\to {\text{NO}}_{3}^{-}+2{\text{H}}^{\text{ + }}+2{\text{e}}^{-}$$

First, nitrite loses an electron to form NO_2 _[reaction (1)]. Second, this step is followed by a homogeneous disproportionation [reaction (2)] of NO_2 _into nitrate and nitrite, which can be written as the total reaction (3) [45,46].

Regarding the oxidation peak of ${\text{NO}}_{2}^{-}$ at CuO/GCE, the potential scan rate was investigated clearly as shown in Figure 5(B). The peak current is proportional to the square root of scan rate in the range of 10-200 mV/s, i_pa_/μA = -3.5542 - 1.9517 v^1/2^/mV·s^-1^, R = 0.9995, while the E_pa _shifted positively. The results suggested that the oxidation of ${\text{NO}}_{2}^{-}$ was undergoing a diffusion controlled process [40].

**Figure 6:**
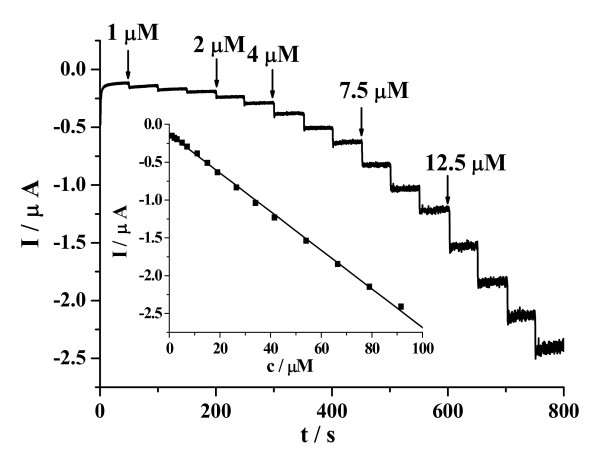
**Amperometric responses of CuO/GCE upon the successive addition of ${\text{NO}}_{2}^{-}$ into gently stirred 0.1M PBS at 0.85V**. Inset: the linear relationships between the catalytic current and the concentration.

Figure 6 illustrates current-time plots for the CuO/GCE under the optimized experimental conditions with successive step changes of ${\text{NO}}_{2}^{-}$ concentration. As the ${\text{NO}}_{2}^{-}$ was injected into the stirring PBS, the steady-state currents reached another steady-state value (95% of the maximum) in less than 3 s. Such a fast response implies that the CuO can promote the oxidation of ${\text{NO}}_{2}^{-}$. The linear relationship between the catalytic current and the concentration is shown in the inset of the Figure 6. As can be seen, the CuO/GCE displays linear response range of 1.0 × 10^-6 ^to 91.5 ×10^-6 ^M (correlation coefficient: 0.9994), with a detection limit of 3.6 ×10^-7 ^M at a signal-to-noise ratio of 3 and a sensitivity of -25.53 μA/mM. Note that the obtained detection limit of 3.6 ×10^-7 ^M is comparable or lower than those obtained with other electrochemical methods [30,31,47-50]. We have summarized various ${\text{NO}}_{2}^{-}$ sensors in Table 2 with respect to the linear range and the detection limit. Compared with other reported values [30,31,47-50] in Table 2, CuO/GCE exhibits the comparable or lower detection limit but narrower linear range.

**Table 2:** Comparison of performances of different electrochemical sensors for nitrites.

Electrode	Linear range (μM)	Detection limit (μM)	Reference
Nano-Au/Ch/GC	0.4-750	0.1	[47]
Nano-Au/P3MT/GC	5-500	2.3	[48]
Pt disk electrode	10-10 000	4.8	[49]
Cu-Tl composite film-GCE	1000-10 000	250	[50]
CuO-Graphite Composite EC	100- 1 250	0.6	[30]
MnO_2_-Graphite Composite EC		1.2	[31]
CuO-GCE	1.0-91.5	0.36	This work

The potential interference for the detection of nitrite using this electrochemical sensor was also examined by adding the following ions into the PBS solution at the same concentration that was used for nitrite: K^+^, Na^+^, Mg^2+^, Zn^2+^, Cl^-^, ${\text{SO}}_{4}^{2-}$ and ${\text{PO}}_{4}^{3-}$. None of the ions caused interference. The potential of nitrite oxidation is high and, as a result, other electroactive species present in complex like dopamine and ascorbic acid can in principle be oxidized as well, interfering with the nitrite analysis. However, the detection of nitrite was examined in presence of 20-fold amount of dopamine and ascorbic acid and showed no interference. This result is similar to that of the previous report [30], which demonstrates the selectivity of CuO based electrochemical sensor towards nitrite.

## Conclusions

In summary, flower like CuO composed of many nanoflake with average thickness of 40 nm was synthesized by a simple hydrothermal reaction. Then a novel electrochemical sensor made of CuO onto GCE had been constructed. Due to the large specific surface area and inner characteristic of the flower like CuO, the resulting electrode show excellent electrocatalytic reduction for H_2_O_2 _and oxidation of ${\text{NO}}_{2}^{-}$. Its sensitivity, low detection limit, fast response time and simplicity are satisfactory. Furthermore, this synthetic approach can also be applied for the synthesis of other inorganic oxides with improved performances and they can also be extended to construct other micro/nano-structured functional surfaces.

## Competing interests

The authors declare that they have no competing interests.

## Authors' contributions

LZ completed the electrochemical work, data treatment and drafted the manuscript. FY participated in data analysis. XZ carried out the work of preparation, participated in data collection. LY participated in data analysis. All authors have read and approved the final manuscript.
